# Breaking the barriers in cancer care: The next generation of herpes simplex virus-based oncolytic immunotherapies for cancer treatment

**DOI:** 10.1016/j.omto.2023.100729

**Published:** 2023-09-19

**Authors:** Nikhil I. Khushalani, Kevin J. Harrington, Alan Melcher, Praveen K. Bommareddy, Dmitriy Zamarin

**Affiliations:** 1 Moffitt Cancer Center, Tampa, FL, USA; 2 The Institute of Cancer Research, London, UK; 3 Replimune, Inc., Woburn, MA, USA; 4 Icahn School of Medicine at Mount Sinai, New York, NY, USA

**Keywords:** combination therapy, HSV, checkpoint inhibitors, genome engineering, immunotherapy, oncolytic therapy

## Abstract

Since the US Food and Drug Administration first approved talimogene laherparepvec for the treatment of melanoma in 2015, the field of oncolytic immunotherapy (OI) has rapidly evolved. There are numerous ongoing clinical studies assessing the clinical activity of OIs across a wide range of tumor types. Further understanding of the mechanisms underlying the anti-tumor immune response has led to the development of OIs with improved immune-mediated preclinical efficacy. In this review, we discuss the key approaches for developing the next generation of herpes simplex virus-based OIs. Modifications to the viral genome and incorporation of transgenes to promote safety, tumor-selective replication, and immune stimulation are reviewed. We also review the advantages and disadvantages of intratumoral versus intravenous administration, summarize clinical evidence supporting the use of OIs as a strategy to overcome resistance to immune checkpoint blockade, and consider emerging opportunities to improve OI efficacy in the combination setting.

## Graphical abstract

**Figure:**
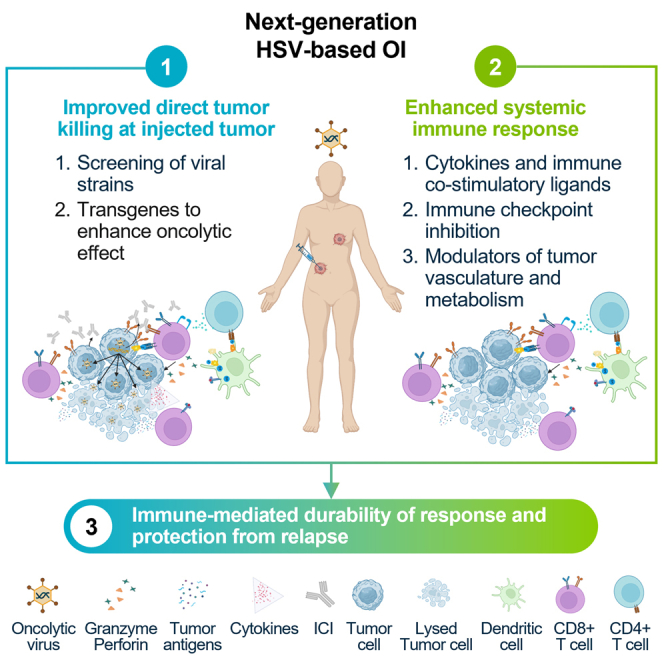

## Teaser

Based on the experience of T-VEC and mechanistic studies exploring anti-tumor immune responses, the field of HSV-based OI has expanded rapidly, now targeting diverse tumor types and clinical indications. This review summarizes the rationale and strategies behind development of these next-generation HSV-based OIs, with a focus on clinical evidence.

## Introduction

Oncolytic immunotherapies (OIs) can be broadly characterized based on virus type (DNA or RNA), genome size, tropism, mechanism of cellular entry, and immunogenicity.^1^ They are either native or genetically modified such that they enter, replicate in, and kill tumors without damaging normal tissue.^2^ Local tumor killing by OIs leads to release of tumor-associated antigens (including neoantigens), viral pathogen-associated molecular patterns, and cell-derived damage-associated molecular patterns necessary for induction of a systemic anti-tumor immune response.^2^^,^^3^ Most OIs are also capable of carrying transgenes, the expression of which can aid in further engagement of innate and adaptive immunity by directing the production of cytokines, antibodies capable of binding to immune checkpoint molecules, and/or other immune stimulatory proteins locally in the tumor microenvironment.^4^ The ability of OIs to turn “cold” tumor microenvironments “hot,” paired with enhanced recruitment of cytotoxic lymphocytes, may further increase the susceptibility of tumors to immune checkpoint inhibitors (ICIs), which are otherwise largely ineffective in non-immune-infiltrated tumors.^5^ Most clinically utilized OIs are heavily attenuated viruses or naturally occurring, less virulent variants that exhibit tumor selectivity while limiting potential unwanted toxicity.^2^ The majority of the reported adverse events are self-limiting and do not overlap with those of conventional cancer treatments such as chemotherapy and radiotherapy.^6^^,^^7^ Thus, given the mechanistic rationale for synergistic potential, excellent safety profile, and off-the-shelf utility, OIs are attractive candidates for combination-based immunotherapeutic approaches.^8^

Two of the earliest oncolytic viruses to receive regulatory approval were Rigvir (ECHO-7 virus) in 2004 in Latvia for the treatment of melanoma and Oncorine (H101; adenovirus) in 2005 in China for the treatment of nasopharyngeal carcinoma.^9^^,^^10^ Although well tolerated, limited data exist demonstrating efficacy of these first-generation oncolytic viruses.^5^ Talimogene laherparepvec (T-VEC), a herpes simplex virus type 1 (HSV-1) that encodes granulocyte-macrophage colony-stimulating factor (GM-CSF), was designed to enhance anti-tumor immune response in addition to direct oncolytic effect.^4^ T-VEC was approved by the US Food and Drug Administration (FDA) in 2015 for treatment of advanced and recurrent melanoma.^4^^,^^11^ Given that HSV-based T-VEC is the only approved OI in the US and several additional HSV-based OIs have reached advanced stages of clinical development, this review aims to summarize the current clinical landscape of this next generation of OIs specifically highlighting OIs based on the HSV platform.

## General considerations for selection of a virus species for use as an OI

When selecting a virus species, there are several structural and functional differences among viruses for consideration. For example, the pathogenicity among viruses is highly variable, which influences decisions regarding whether engineering or other strategies are needed.^1^^,^^12^ As an additional safety feature, some virus species used as the basis for OI development are susceptible to readily available antiviral therapies.^2^ Virus selection might also be influenced by the site of viral replication, such as the cytoplasm versus the nucleus, as cytoplasmic replication limits any potential concerns for insertional mutagenesis.^2^^,^^13^ Where needed, engineering strategies can, therefore, allow production of viral strains that replicate selectively in tumors.^12^^,^^14^ Finally, the capacity of the virus to carry transgenes is also a key consideration, which is influenced by both the size of the viral genome and the type of capsid of the underlying virus species.^15^

Naturally occurring viruses that replicate selectively in tumors, as compared to normal tissues, include reovirus, Newcastle disease virus, parvovirus H-1, alpha virus M1, coxsackievirus, and various picornaviruses.^6^ Virus species that can be engineered or otherwise attenuated to provide tumor-selective replication, and that can also be engineered to encode transgenes to enhance anti-tumor efficacy, include adenoviruses, retroviruses, herpes virus, poxviruses, vesicular stomatitis virus, and measles virus.^6^^,^^16^ Many of these are being tested in clinical studies as potential candidate OIs.^6^^,^^16^ Adenoviruses are nonenveloped, double-stranded DNA (dsDNA) viruses with modestly sized genomes that can accommodate additional transgenes and have been studied extensively in preclinical and clinical studies.^7^^,^^17^ HSV (HSV-1 and HSV-2) is a large dsDNA virus with broad lytic ability, and its genome can accommodate substantial additional DNA (∼20–30 kb after deletion of genes to provide tumor selectivity) for transgene insertion.^13^ Replication of HSV occurs in the nucleus but has not been linked to insertional mutagenesis.^18^ Vaccinia virus (an attenuated poxvirus) and coxsackievirus have replication cycles occurring entirely in the cytoplasm of host cells, again limiting concerns regarding insertional mutagenesis.^19^^,^^20^ Vaccinia virus has a large dsDNA genome (∼190 kb), is highly tumor-tropic, and is relatively innocuous in immunocompetent people.^2^ Coxsackievirus is a nonenveloped single-stranded RNA enterovirus that can induce a strong immune reaction^21^^,^^22^; however, there is limited space for the insertion of transgenes in the coxsackievirus genome, thus restricting the engineering potential of this virus.^22^ Measles viruses are small-genome (∼16 kb) RNA paramyxoviruses that also replicate within the cytoplasm and are capable of spreading through cell-to-cell fusion, a potential advantage for OI spread throughout the tumor.^23^

## Strategies to increase direct tumor-killing effects

Several strategies to increase the natural ability of OIs to selectively kill tumors have been developed. One approach is to incorporate “suicide genes” (i.e., genes that increase the susceptibility of cells to exogenously provided prodrugs or that otherwise induce cell death) directly into the OIs.^2^ These genes can include enzymes that increase the cytotoxicity or convert otherwise benign prodrugs into potent cytotoxic agents only in cells expressing the suicide gene. For example, viral vectors can deliver the HSV-thymidine kinase (TK) gene,^24^ which converts thymidine analogues such as ganciclovir into monophosphates. These, in turn, get further phosphorylated by cellular kinases into triphosphates that are incorporated into the DNA of replicating cancer cells, causing a termination of DNA synthesis and subsequent cell death.^25^ Other examples of suicide genes include cytosine deaminase, which functions similarly to TK, and adenovirus death protein, which enhances cell lysis.^26^^,^^27^

**Figure 1:**
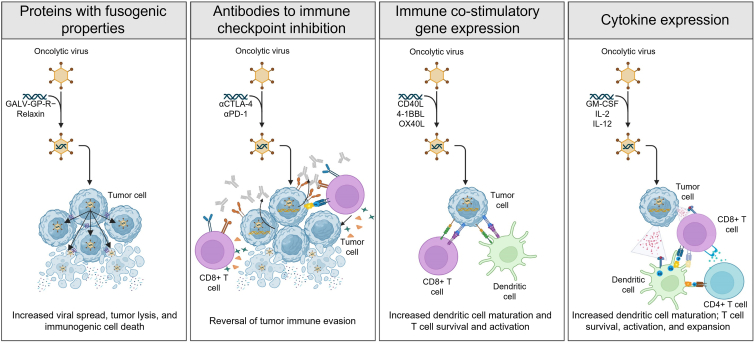
Genetic engineering approaches to improve OI efficacy The therapeutic efficacy of OIs can be improved by the insertion of transgenes into the viral vector. These transgenes can directly influence the viral spread or lytic properties of the OI (e.g., GALV-GP-R− and relaxin), block immune checkpoint inhibition (e.g., αCTLA-4 and αPD-1), provide immune co-stimulatory signals (e.g., CD40L, 4-1BBL, and OX40L), and induce proinflammatory cytokine secretion (e.g., GM-CSF, IL-2, and IL-12). Collectively, the goal of these transgenes is to improve systemic anti-tumor immune responses and reverse the immunologically cold tumor microenvironment. 4-1BBL, 4-1BB ligand; αCTLA-4, anti-cytotoxic T-lymphocyte antigen 4; αPD-1, anti-programmed cell death protein 1; CD40L, cluster of differentiation 40 ligand; CD8, cluster of differentiation 8; GALV-GP-R−, gibbon ape leukemia virus glycoprotein with the R sequence deleted; GM-CSF, granulocyte-macrophage colony-stimulating factor; IL, interleukin; OI, oncolytic immunotherapy; OX40L, OX40 ligand.

Fusogenicity is another feature of some OIs, such as the measles virus, that enhances oncolytic activity through improved tumor killing and virus spread via cell-to-cell fusion.^28^ Enhancement of fusogenic properties of other viruses through insertion of genes coding for fusogenic proteins has recently been demonstrated as an approach to improve oncolytic activity.^14^^,^^28^ Expression of fusogenic glycoproteins on the surface of an infected cell induces cell-to-cell fusion,^29^ resulting in formation of nonviable, multinucleated giant cells, known as syncytia.^29^ In addition, by spreading through direct cell-to-cell spread, a neutralizing antibody response against the OI may be avoided. Insertion of the gibbon ape leukemia virus (GALV) fusogenic protein into HSV-1 augmented tumor killing in preclinical models and, in clinical trials, led to the presence of higher copies of viral DNA circulating in the blood as compared to prior HSV-1-based OIs, suggesting that viral replication was also increased.^14^^,^^30^^,^^31^^,^^32^ Syncytium formation is also associated with immunogenic cell death, which potentiates anti-tumor immune responses through promoting antigen presentation by dendritic cells.^28^^,^^33^ In addition to GALV, other viral fusion proteins have been incorporated into OIs, such as the reovirus fusion-associated small transmembrane proteins.^29^^,^^34^^,^^35^ Figure 1 provides an overview of how the different transgenes included in these OIs can influence efficacy by acting directly upon the tumor itself or influencing the tumor microenvironment. While the optimal engineering strategies and therapeutic transgenes are not known, the selection of specific therapeutic transgenes should be based on the underlying tumor biology or clinical indication. For example, for tumors characterized by an immune-excluded tumor microenvironment, secondary to a lack of antigen presentation or suboptimal immune priming, engineering strategies targeted toward activation of antigen-presenting cells and T cell co-stimulation may be indicated.^36^^,^^37^ The inclusion of transgenes that drive virus-mediated cell death also has the potential to achieve greater tumor debulking and further drive the mode of killing toward immunogenic cell death. Due to safety concerns, some therapeutic transgenes can only be delivered via direct intratumoral injection; thus, based on the clinical setting (e.g., intratumoral versus systemic OI delivery), specific transgenes may be chosen on the basis of the perceived safety profile. As this relatively new field evolves, emergent data will yield insights into the identification of optimal transgenes for incorporation into the next generation of OIs.

## Limiting virulence and toxicity

Tumor selectivity of OIs relies on several biological characteristics of tumors, including expression of viral entry receptors, dysregulation of signaling and antiviral pathways, and altered metabolism.^38^ For example, the herpes virus entry mediator Nectin-1 is overexpressed in some cancer cells, including melanoma.^39^ The rapid rate of cellular division and high metabolic activity of tumors may also support increased viral replication compared to normal tissue.^38^ Probably most critically, deficiencies in antiviral interferon (IFN) signaling, which is a cardinal feature of many tumors that helps evade host cellular tumor suppressor mechanisms, support OI replication specifically in tumors.^40^ Conversely, competent type I IFN signaling in normal tissues helps limit off-target effects of OIs.^41^

In addition to capitalizing on the natural preferential ability of OIs to replicate in tumors, several mechanisms to further reduce viral replication in non-tumor tissue have been explored. These include the use of tumor tissue-specific promoters for expression of viral genes (e.g., human telomerase reverse transcriptase,^17^ MYB-related protein B,^42^ prostate-specific antigen,^43^ and E2F^4^^,^^17^) as well as microRNA (miRNA)-based approaches, where tissue-specific miRNA targets are inserted into OI essential genes to restrict viral replication in that tissue.^44^ Examples include targeting of infected cell protein (ICP)-4, ICP27, or UL8, which are essential for viral replication.^44^ Additional viral modifications using deletions or miRNA-mediated suppression of virulence factors can be generated.^2^ For example, with HSV, ICP34.5 is a critical viral protein that inhibits host antiviral responses and is necessary for neurovirulence.^45^ Its deletion generates a non-neurovirulent virus, which is non-pathogenic in humans.^45^^,^^46^ ICP47, another HSV virulence factor, inhibits antigen presentation and thus limits viral detection by lymphocytes, prolonging viral infection.^47^ Consequently, deletion of ICP47 improves virus (and likely also tumor) recognition by T cells, thereby reducing viral spread in healthy tissue.^47^ Deletion of ICP34.5 and ICP47 was the strategy used for attenuation of T-VEC. As a testament to the success of this design, the phase 3 OPTiM trial demonstrated T-VEC was safe, and no reactivation of latent infection by T-VEC was reported.^31^^,^^48^

## HSV-1-based OIs

**Figure 2:**
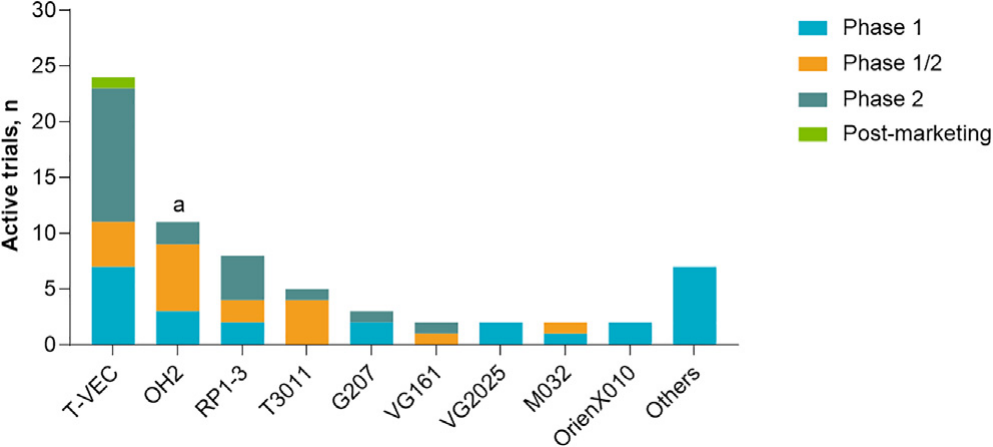
HSV-based OIs in clinical trials A preliminary search of ClinicalTrials.gov was performed on March 8, 2023, using the following search terms: (HSV OR “herpes simplex virus”) AND (oncolytic or tumor) and filtered by clinical trial status (recruiting; not yet recruiting; active, not recruiting; and enrolling by invitation). After the preliminary search, the names of identified agents were then used for a secondary search. No active phase 3 studies were identified on ClinicalTrials.gov. ^a^OH2 is an HSV-2-based OI. T-VEC, RP1-3, T3011, G207, VG161, VG2025, M032, and OrienX010 are based on HSV-1 platforms. HSV, herpes simplex virus; OI, oncolytic immunotherapy; T-VEC, talimogene laherparepvec.

As previously discussed, among virus species explored for use as OIs, HSV-1 has emerged as one of the most widely studied and serves as the vector backbone for T-VEC.^49^^,^^50^ HSV is an ideal candidate for the development of OIs for many reasons. First, HSV has proven safety data and clinical activity, as reviewed in greater detail below. Second, the base genome is relatively easy to genetically manipulate and has the capacity to accept multiple large transgenes. In addition, it is amenable to genetic engineering approaches to improve safety (e.g., deletion of ICP34.5 and ICP47).^13^ Third, HSV carries minimal risk for recombination into the host genome. Finally, unlike some other viral candidates for OI, there are antivirals available for HSV in the event of unintended exposure.^2^ Accordingly, there are many active clinical trials registered at ClinicalTrials.gov evaluating HSV-based OIs (Figure 2; Table 1). Several examples of these HSV-1-based OIs are discussed next, including novel platforms with emerging data, reflecting the rapidly evolving landscape of OIs.

**Table 1:** HSV-based OIs in phase 1/2 or later clinical trials

Agent name	Trial description	Study phase	Injection type	Monotherapy vs. combination	NCT #
T-VEC	T-VEC and preoperative radiation for sarcoma	phase 1/2	intratumoral	combination	NCT04599062
	T-VEC in combination with neoadjuvant chemotherapy in triple-negative breast cancer	phase 1/2	intratumoral	combination	NCT02779855
	T-VEC and preoperative radiation for sarcoma	phase 1/2	intratumoral	combination	NCT02453191
	T-VEC injected into tumors alone and in combination with systemic pembrolizumab MK-3475-611/KEYNOTE-611	phase 1/2	intratumoral	monotherapy/combination	NCT02509507
	T-VEC in patients with cutaneous squamous cell cancer	phase 2	intratumoral	monotherapy	NCT03714828
	T-VEC in classic or endemic Kaposi sarcoma	phase 2	intralesional	monotherapy	NCT04065152
	neoadjuvant T-VEC in high-risk early melanoma	phase 2	intralesional	monotherapy	NCT04427306
	T-VEC in combination with pembrolizumab in patients with metastatic and/or locally advanced sarcoma	phase 2	intralesional	combination	NCT03069378
	neo-adjuvant T-VEC + nivolumab combination therapy for resectable early metastatic (stage IIIB/C/D-IV M1a) melanoma with injectable disease	phase 2	intralesional	combination	NCT04330430
	T-VEC and pembrolizumab in treating patients with stage III–IV melanoma	phase 2	intralesional	combination	NCT02965716
	T-VEC, nivolumab and trabectedin for sarcoma	phase 2	intratumoral	combination	NCT03886311
	T-VEC and radiation therapy in treating patients with newly diagnosed soft tissue sarcoma that can be removed by surgery	phase 2	intratumoral	combination	NCT02923778
	T-VEC and nivolumab in treating patients with refractory lymphomas or advanced or refractory non-melanoma skin cancers	phase 2	intratumoral	combination	NCT02978625
	neoadjuvant combination immunotherapy for stage III melanoma	phase 2	intralesional	combination	NCT03842943
	T-VEC with pembrolizumab in melanoma following progression on prior anti-PD-1-based therapy (MASTERKEY-115; MK-3475-A07/KEYNOTE-A07)	phase 2	intralesional	combination	NCT04068181
	T-VEC with or without radiotherapy for melanoma, Merkel cell carcinoma, or other solid tumors	phase 2	intralesional	monotherapy/combination	NCT02819843
OH2	OH2 oncolytic viral therapy in non-muscle-invasive bladder cancer	phase 1/2	intravesical	monotherapy	NCT05232136
	OH2 oncolytic viral therapy in central nervous system tumors	phase 1/2	intracavity	monotherapy	NCT05235074
	OH2 oncolytic viral therapy in pancreatic cancer	phase 1/2	intratumoral	monotherapy	NCT04637698
	OH2 injection in combination with HX008 for melanoma	phase 1/2	not specified	combination	NCT04616443
	OH2 oncolytic viral therapy in solid tumors	phase 1/2	intratumoral	monotherapy/combination	NCT03866525
	OH2 injection in solid tumors	phase 1/2	not specified	monotherapy/combination	NCT04386967
	OH2 oncolytic viral therapy in advanced bladder cancer	phase 2	intratumoral	monotherapy	NCT05248789
	first-line maintenance of OH2 injection for advanced colorectal cancer	phase 2	intratumoral	combination	NCT05648006
RP1	RP1 in solid organ and hematopoietic cell transplant recipients with advanced cutaneous malignancies	phase 1/2	intratumoral	monotherapy	NCT04349436
	RP1 as a single agent and in combination with PD-1 blockade in patients with solid tumors	phase 1/2	intratumoral	monotherapy/combination	NCT03767348
	cemiplimab as a single agent and in combination with RP1 in patients with advanced cutaneous squamous cell carcinoma	phase 2	intratumoral	combination	NCT04050436
RP2/3	RP2/RP3 OI in combination with atezolizumab and bevacizumab for the treatment of patients with advanced microsatellite-stable and mismatch repair-proficient colorectal carcinoma	phase 2	intratumoral	combination	NCT05733611
RP3	RP3 OI in combination with first- or second-line therapy in patients with locally advanced unresectable or metastatic hepatocellular carcinoma	phase 2	intratumoral	combination	NCT05733598
	RP3 OI in combination with other therapy in patients with locoregionally advanced or recurrent squamous cell carcinoma of the head and neck	phase 2	intratumoral	combination	NCT05743270
T3011	T3011 administered intravenously in patients with advanced solid tumors	phase 1/2	intravenous	monotherapy	NCT05598268
	T3011 administered via intratumoral injection in patients with advanced solid tumors	phase 1/2	intratumoral	monotherapy	NCT05602792
	T3011 given as a single agent and in combination with other therapy in subjects with advanced solid tumors	phase 1/2	intravenous	monotherapy/combination	NCT04780217
	T3011 given as a single agent and in combination with intravenous pembrolizumab in participants with advanced or metastatic solid tumors	phase 1/2	intratumoral	monotherapy/combination	NCT04370587
	T3011 in combination with cobimetinib in patients with advanced melanoma	phase 2	intratumoral	combination	NCT05756556
G207	G207 with a single radiation dose in children with recurrent high-grade glioma	phase 2	intratumoral	monotherapy	NCT04482933
VG161	VG161 in combination with nivolumab in subjects with advanced pancreatic cancer	phase 1/2	intratumoral	combination	NCT05162118
	VG161 in the treatment of patients with hepatocellular carcinoma or intrahepatic cholangiocarcinoma	phase 2	intratumoral	monotherapy	NCT05223816
M032	pembrolizumab and M032	phase 1/2	not specified	combination	NCT05084430

Results are only shown for active phase 1/2 or later-stage clinical trials that use HSV-based OIs registered at ClinicalTrials.gov. A preliminary search of ClinicalTrials.gov was performed on March 8, 2023, using the following search terms: (HSV OR “herpes simplex virus”) AND (oncolytic or tumor) and filtered by clinical trial status (recruiting; not yet recruiting; active, not recruiting; and enrolling by invitation). After the preliminary search, the names of identified agents were then used for a secondary search.

HSV, herpes simplex virus; OI, oncolytic immunotherapy; PD-1, programmed cell death protein 1; T-VEC, talimogene laherparepvec.

### T-VEC

**Figure 3:**
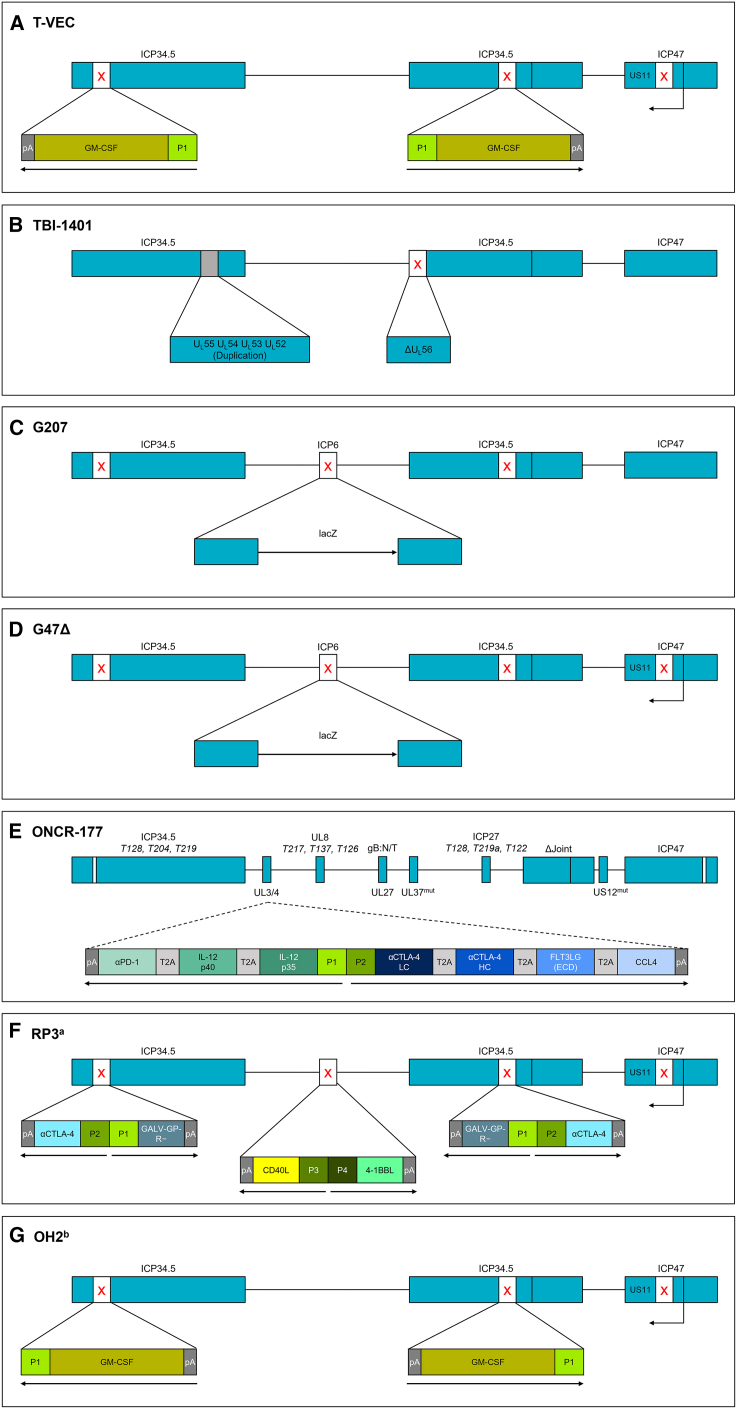
Example HSV-based OIs in clinical development (A) T-VEC, (B) TBI-1401, (C) G207, (D) G47Δ, (E) ONCR-177, (F) RP3, and (G) OH2. The OI backbone schematics were constructed based on the descriptions provided in the following reports: Hu et al.,^54^ Eissa et al.^55^ (TBI-1401), Cripe et al.^56^ (G207), Todo et al.^12^ (G47Δ), Haines et al.^57^ (ONCR-177), Harrington et al.^58^ (RP3), and Zhang et al.^59^ (OH2). For T3011, while a description of the transgenes included in the OI is published, the genetic modifications to improve the safety of the OI are not reported and so T3011 was not included in the figure. The red “X” denotes deletions to the viral genome. ^a^The RP1–3 platforms of OIs are derived from the RP1 backbone. RP1 contains GALV-GP-R− and human GM-CSF insertions; RP2 contains an additional insertion of αCTLA-4; RP3 has GM-CSF removed and contains additional insertions of 4-1BBL and CD40L. ^b^OH2 is an HSV-2-based OI. 4-1BBL, 4-1BB ligand; αCTLA-4, anti-cytotoxic T-lymphocyte antigen 4; αPD-1, anti-programmed cell death protein 1; CCL4, C-C motif chemokine ligand 4; CD40L, cluster of differentiation 40 ligand; FLT3LG(ECD), extracellular domain of Fms-related receptor tyrosine kinase 3 ligand; GALV-GP-R−, gibbon ape leukemia virus glycoprotein with the R sequence deleted; gB:N/T, mutation of amino acids D285N and A549T in fusogenic glycoprotein B (gB); GM-CSF, granulocyte-macrophage colony-stimulating factor; HC, heavy chain; HSV-1, herpes simplex virus type 1; ICP, infected cell protein; Joint, segment between the unique long and short regions; IL, interleukin; LacZ, bacterial beta-galactosidase; LC, light chain; Mut, mutant; OI, oncolytic immunotherapy; P, promoter; pA, polyadenylation signal; T, miRNA target cassette; T2A, Thosea asigna virus 2A peptide; T-VEC, talimogene laherparepvec; UL, unique long region; US, unique short region.

T-VEC (Imlygic) is a genetically modified HSV-1 deleted for ICP34.5 and ICP47 and expressing GM-CSF (Figure 3A) and is the only OI approved by the FDA and European Medicines Agency.^6^^,^^11^ T-VEC has demonstrated clinical efficacy and tolerability in both clinical trials and in real-world settings.^46^^,^^48^^,^^51^^,^^52^ In the final analysis of the phase 3 OPTiM trial, patients with unresectable stage IIIB-IVM1c melanoma treated with T-VEC achieved improved objective response rate (ORR; 31.5% versus 6.4%), durable response rate (19.3% versus 1.4%), and median survival (23.3 versus 18.9 months) compared with patients receiving GM-CSF therapy.^46^ Translational studies from an open-label phase 2 trial showed that T-VEC increased the intratumoral density of CD8+ and CD4+ lymphocytes and other immune cell infiltrates in both injected and non-injected lesions in patients with stage IIIB-IVM1c melanoma.^53^

Additional trials of T-VEC in other clinical settings have also shown encouraging efficacy. A phase 2 study in resectable melanoma showed a 25% reduction in the risk of disease recurrence with the addition of neoadjuvant T-VEC to surgery versus surgery alone.^60^ In a phase 1/2 study, T-VEC in combination with chemoradiotherapy produced an ORR of 82.3% in patients with squamous cell carcinoma of the head and neck (SCCHN).^61^ Additionally, findings from a phase 2 study demonstrated that T-VEC plus ipilimumab (anti-cytotoxic T-lymphocyte antigen 4 [CTLA-4]) led to improved ORR versus ipilimumab alone (38.8% versus 18.0%, respectively) in patients with advanced, unresectable melanoma.^62^

Additional clinical trials with T-VEC in a number of cancer types have been completed, including trials of T-VEC in combination with immune checkpoint blockade. The MASTERKEY-265 trial evaluated T-VEC in combination with pembrolizumab in advanced melanoma and included a translational component to evaluate changes in cytotoxic T cells induced by combination therapy.^3^ Biomarker data obtained from the phase 1b portion of the trial (n = 21) suggested that, in addition to the observed ORR of 61.9%, the combination of T-VEC and pembrolizumab favorably altered the tumor microenvironment and contributed to systemic anti-tumor effects (e.g., an increase in circulating and intratumoral CD8+ T cells).^3^ Additionally, long-term follow-up from the phase 1b trial demonstrated that median progression-free survival (PFS) and overall survival were still not reached at nearly 5 years of follow-up.^63^ In the randomized, placebo-controlled phase 3 portion of the trial, while the combination resulted in a numerically improved ORR (48.6% versus 41.3%), complete response rate (17.9% versus 11.6%), and PFS (hazard ratio, 0.86; 95% confidence interval, 0.71–1.04) versus placebo plus pembrolizumab, the study did not reach its primary endpoints of PFS and overall survival.^64^ Although subgroup analysis showed benefit in PFS for some populations (e.g., patients enrolled in the US, patients with lactate dehydrogenase ≤ the upper limit of normal, and those with a sum of the longest diameters of target lesions at baseline ≤ the median),^64^ the study was not designed to evaluate differences in these patient populations and the results overall were disappointing. The factors responsible for the discrepancy between the phase 1 and phase 3 results are not entirely clear but could be in part due to differences in various aspects of trial design or execution.^64^^,^^65^ For example, the study design was different between the two trial phases; in the phase 1b portion, administration of T-VEC began 5 weeks before the initiation of pembrolizumab, which was intended to induce an anti-tumor immune response before the addition of pembrolizumab, whereas, in the phase 3 portion, administration of both agents began simultaneously. It is possible that the sequence of T-VEC administration before ICIs is important for priming the tumor microenvironment in order to achieve maximal efficacy.^65^ Prior studies demonstrated that the benefit of T-VEC is most notable among patients with in-transit or limited metastatic disease.^46^^,^^66^ Patients with stage IVMb/IVMc disease accounted for over 50% of enrolled patients in MASTERKEY-265,^64^ suggesting that the advanced disease burden could have contributed to diminished responses to combination therapy.^66^ Contrary to this hypothesis, however, a sensitivity analysis of overall survival in the primary MASTERKEY-265 study demonstrated no difference between treatment arms after excluding patients with stage IVM1c disease, further complicating interpretation of the effect of disease burden.^64^ Furthermore, poor activity of the combination in patients with visceral disease in MASTERKEY-265 should also be considered, particularly given the emerging data demonstrating the immunosuppressive nature of some metastatic tumor microenvironments, such as the liver.^36^^,^^37^ In this regard, many of the subsequent OIs developed since T-VEC, and reviewed below, include the addition of transgenes designed to reverse the immunologically suppressive tumor microenvironment and are currently being evaluated using direct injection into visceral lesions.

Finally, disappointing results were also seen in the MASTERKEY-232 phase 1b/3 trial evaluating T-VEC in combination with pembrolizumab in recurrent or metastatic SCCHN.^67^ While the combination was well tolerated, the phase 1b portion of the trial demonstrated that the efficacy of combination treatment was similar to that seen historically with pembrolizumab monotherapy; of note, the enrolled patient population was arguably more advanced and more frail given early symptomatic progression in a large proportion of patients compared to the historical studies in that setting.^67^

### TBI-1401

TBI-1401 was originally called HF10 because it was derived from the HSV-1 strain HF as clone 10.^55^ While TBI-1401 has several natural deletions and insertions (Figure 3B), the functions of these alterations to the base HSV-1 genome are unclear. TBI-1401 has been evaluated in multiple phase 1 trials for breast cancer, pancreatic cancer, melanoma, and SCCHN.^55^ Additionally, a phase 2 trial evaluated TBI-1401 in combination with anti-CTLA-4 in patients with unresectable or metastatic melanoma (NCT02272855). From the 44 patients with efficacy results in the phase 2 trial, the best overall response rate per immune-related response criteria at 24 weeks was 41% (16% complete response; 25% partial response); 68% achieved disease control (27% had stable disease).^68^ Unlike the other HSV-based OIs, TBI-1401 does not contain any inserted transgenes or engineered genetic modifications to delete viral proteins, highlighting the apparent immunogenic potential of the HSV-1 backbone even without additional immune-activating transgenes. A phase 1 study of TBI-1401 in combination with chemotherapy in patients with unresectable pancreatic cancer is ongoing (NCT03252808).

### G207

G207 is a double-mutated HSV-1, with deletion of ICP34.5 and insertion of *LacZ* to inactivate ICP6 (Figure 3C).^69^^,^^70^ A phase 1 trial evaluated G207 in children with recurrent glioma. While, at recurrence, the median life expectancy of such patients historically is 5.6 months,^71^ the median overall survival after G207 treatment was 12.2 months, with four of the 11 participants who responded to treatment still alive at 18 months.^69^ Several of the participants had pre- and post-treatment biopsy samples. Consistent with other reports of pediatric high-grade glioma, the pre-treatment biopsies had few tumor-infiltrating lymphocytes. However, biopsy tissue obtained 2 to 9 months post treatment demonstrated substantial increases in CD3+, CD4+, and CD8+ tumor-infiltrating lymphocytes.^69^ Based on these encouraging results, a phase 2 clinical trial of G207 with radiation in pediatric high-grade glioma (NCT04482933) is forthcoming.

### G47Δ

G47Δ (Teserpaturev) is a third-generation, triple-mutated (deleted for ICP34.5, ICP6, and ICP47) version of HSV-1 developed from G207 (Figure 3D).^12^^,^^72^ Phase 1/2 clinical trials in patients with glioblastoma demonstrated safety,^73^^,^^74^^,^^75^ and a phase 2 trial in 19 patients showed a 1-year survival rate of 84.2%.^72^ Based on these data, G47Δ received conditional and time-limited approval in Japan for the treatment of glioma contingent upon verification in follow-up studies^75^ and is also being tested for efficacy in other solid tumors.^74^ Evidence of preclinical synergy of G47Δ with systemic anti-CTLA-4 antibody further supports clinical evaluation of this strategy in patients.^76^

### T3011

T3011 is a genetically modified HSV-1 with the insertion of two transgenes encoding interleukin (IL)-12 and an anti-programmed cell death protein 1 (PD-1) antibody.^77^ However, it is not clear from published reports what genetic modifications have been introduced to T3011 to improve safety. The local secretion of IL-12 is anticipated to stimulate IFN-gamma production within the tumor microenvironment, resulting in increased oncolytic activity from natural killer cells and cytotoxic T lymphocytes. Preliminary data from a phase 1 dose-escalation study in patients with advanced cutaneous or subcutaneous malignancies demonstrated that T3011 was well tolerated, and five out of six evaluable patients achieved stable disease as the best response.^77^ The study is ongoing (NCT04370587).^77^

### ONCR-177

ONCR-177 is a genetically modified oncolytic HSV-1 that retains one copy of ICP34.5 to increase resistance to IFN signaling^44^ and uses miRNA-mediated suppression of key viral genes to provide tumor-specific replication and safety. ONCR-177 also encodes five transgenes intended to boost anti-tumor immunity, including IL-12, Fms-like tyrosine kinase 3 ligand, C-C motif chemokine ligand 4, and antagonists to CTLA-4 and PD-1 (Figure 3E). This combination of transgenes was selected to recruit and activate key cells of the immune system central to effective anti-tumor immunity.^57^ An open-label phase I study with ONCR-177 alone or in combination with pembrolizumab for surface lesions or intrahepatic injection is ongoing (NCT04348916).^78^ However, it was recently announced that further development of ONCR-177 has been discontinued.^79^

### RP1–3

RP1 is a replication-competent, enhanced-potency oncolytic HSV-1. It is constructed using a new clinical strain of HSV-1 (RH018) selected following a comprehensive screen of clinical isolates of HSV-1 for its superior oncolytic activity *in vitro*.^14^ RP1 was further modified to express the fusogenic gibbon ape leukemia virus glycoprotein with the R sequence deleted (GALV-GP-R−) to increase oncolysis and promote immunogenic cell death.^14^ RP1 also expresses a codon-optimized version of human GM-CSF, which regulates multiple aspects of dendritic cell function.^14^ In preclinical models, the addition of the GALV-GP-R− transgene to the HSV-1 backbone improved oncolytic activity and increased the uninjected tumor response compared with the same HSV-1-based OI expressing GM-CSF alone.^14^ Furthermore, GALV-GP-R− drove the mode of cancer killing toward immunogenic cell death as shown by increased release of HMGB1, ATP, and the accumulation of calreticulin on the cell surface (hallmarks of a cell undergoing immunogenic cell death as opposed to apoptosis).^14^ Collectively, the results from preclinical models suggest that the combination of GM-CSF and GALV-GP-R− transgenes in an OI offers a greater ability to directly kill tumors and induce systemic anti-tumor immune responses compared with GM-CSF alone; however, head-to-head comparisons in clinical trials have not been performed.

Preliminary data from the phase 1/2 IGNYTE clinical trial (NCT03767348) of RP1 in combination with nivolumab (anti-PD-1) in patients with skin cancer demonstrated evidence of durable response, with a median (range) duration of response of 13.3 (3.7–16.9) months, including responses in those with cutaneous melanoma who did not respond to or progress on anti-PD-1 or anti-PD-1/anti-CTLA-4 therapy (six out of 16; ORR 37.5%). Durable anti-tumor activity was also observed in patients with anti-PD-1-naive non-melanoma skin cancers with a median (range) duration of response of 7.3 (1.9–23.1) months, with responses observed in cutaneous squamous cell carcinoma (11 out of 17; ORR 64.7%), Merkel cell carcinoma (three out of four; ORR 75.0%), basal cell carcinoma (one out of four; ORR 25.0%), and angiosarcoma (four out of six; ORR 66.7%).^80^ A consistent increase in both CD8+ tumor-infiltrating lymphocytes and PD-L1 expression was observed in patients for whom pre- and post-treatment biopsies were available.^81^ This trial also includes a registration-directed cohort in anti-PD-1-failed melanoma with a target enrollment of 125 patients with locally advanced or metastatic disease. Preliminary data from the first 91 patients with anti-PD-1-failed disease (16 patients from the initial melanoma cohort plus 75 patients from the registration-directed cohort) demonstrated that RP1 plus nivolumab achieved an ORR of 37.4% (18.7% complete response rate), with responses observed in both injected and uninjected lesions.^82^ The initial positive responses observed from the expanded cohort in the IGNYTE trial contrast a similar trial evaluating T-VEC and pembrolizumab in anti-PD-1-failed melanoma in which no confirmed responses were observed in patients with visceral lesions (NCT02965716).^83^ Based on these results, RP1 is also being tested in combination with the anti-PD-1 antibody cemiplimab versus cemiplimab alone in an ongoing, randomized phase 2 trial (NCT04050436).

Building on the RP1 backbone, which was largely designed for intratumoral injection of cutaneous, potentially more immunogenic malignancies, RP2 and RP3 were designed to overcome a more immunosuppressive microenvironment associated with visceral lesions such as the liver. RP2 expresses an anti-CTLA-4-like antibody as a means to optimize immune priming at the tumor site while reducing the risk of immune-related adverse events associated with systemic anti-CTLA-4 therapy.^84^^,^^85^ In a phase 1 clinical trial (NCT04336241), RP2 has demonstrated good tolerability to date, as well as encouraging clinical activity in advanced solid tumors. In an early study in patients who have failed prior anti-PD-1 therapy, RP2 in combination with nivolumab demonstrated an ORR of 44.4% (four out of nine) in cutaneous melanoma, 25.0% (two out of eight) in uveal melanoma, and 33.3% (one out of three) in SCCHN.^58^ RP2 and RP3, discussed below, are also being tested in a phase 2 clinical trial in combination with atezolizumab and bevacizumab in patients with advanced microsatellite-stable and mismatch repair-proficient colorectal carcinoma (NCT05733611).

Advancements in the understanding of immune signals governing tumor immune recognition led to development of RP3, a vector designed to overcome the suppressive microenvironment of immunologically cold tumors. Along with the modifications found in RP2, RP3 expresses a pair of immune co-stimulatory pathway-activating ligands, CD40 ligand and 4-1BB ligand, but does not express GM-CSF (Figure 3F). These features encompass multiple aspects of the innate and adaptive immune response, including antigen-presenting cell activation, T cell co-stimulation, and inflammatory cytokine release.^86^ RP3 is currently being tested with and without nivolumab in a phase 1 clinical trial in patients with solid tumors (NCT04735978) and a phase 2 clinical trial in patients with advanced or recurrent SCCHN (NCT05743270). RP3 is also being evaluated in combination with atezolizumab and bevacizumab in a phase 2 clinical trial in patients with advanced hepatocellular carcinoma not amenable to resection or locoregional therapies (NCT05733598).

## HSV-2-based OIs

**Figure 4:**
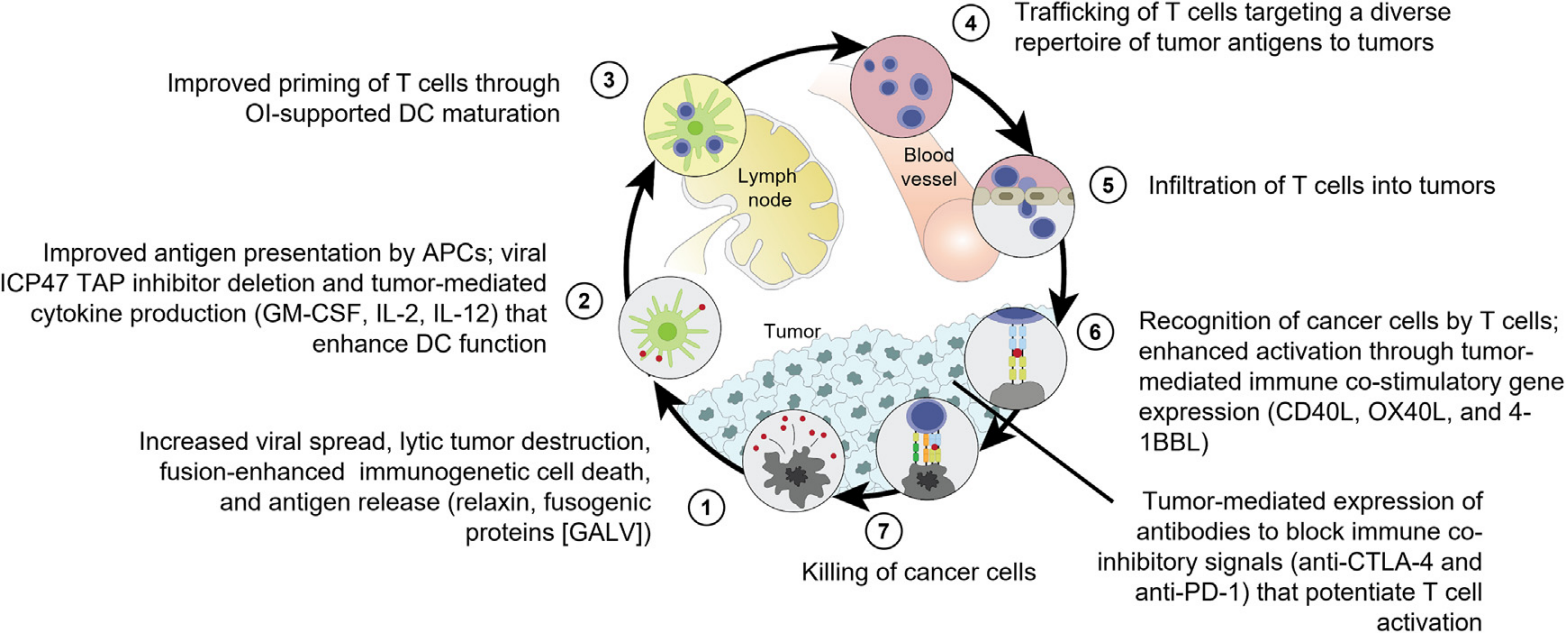
The tumor immunity cycle and opportunities for enhancement through genetic modifications of OIs Adapted from *Immunity*, volume 39, issue 1, Chen DS and Mellman I. Oncology meets immunology: The cancer-immunity cycle, pages 1–10, Copyright 2013, with permission from Elsevier. 4-1BBL, 4-1BB ligand; APC, antigen-presenting cell; CD40L, cluster of differentiation 40 ligand; CTLA-4, cytotoxic T-lymphocyte antigen 4; DC, dendritic cell; GALV, gibbon ape leukemia virus glycoprotein; GM-CSF, granulocyte-macrophage colony-stimulating factor; ICP47, infected cell protein 47; OI, oncolytic immunotherapy; OX40L, OX40 ligand; PD-1, programmed cell death protein 1; TAP, transporter associated with antigen processing.

While the majority of HSV-based OIs are derived from HSV-1, development of other OIs based on HSV-2 is also ongoing. Among these, OH2 is derived from the wild-type HSV-2 strain HG52 and is the furthest along in terms of clinical development. Like many of the OIs discussed above, OH2 has the ICP34.5 and ICP47 genes deleted and encodes GM-CSF to boost anti-tumor immunity (Figure 3G). A phase 1/2 trial of OH2 alone and in combination with an anti-PD-1 antibody (HX008) demonstrated an early signal of activity.^59^ Of the 40 patients treated with OH2 monotherapy and the 14 patients treated with the combination therapy, four patients achieved partial responses, with two from each cohort.^59^ OH2 in combination with anti-PD-1 therapy is being tested in several cancer types, including melanoma (NCT04386967). Figure 4 depicts the multifaceted mechanism of action for OIs, and the differential properties of the HSV-based therapies described above are provided in Table S1.

## Combination of OI with other cancer treatment strategies

**Figure 5:**
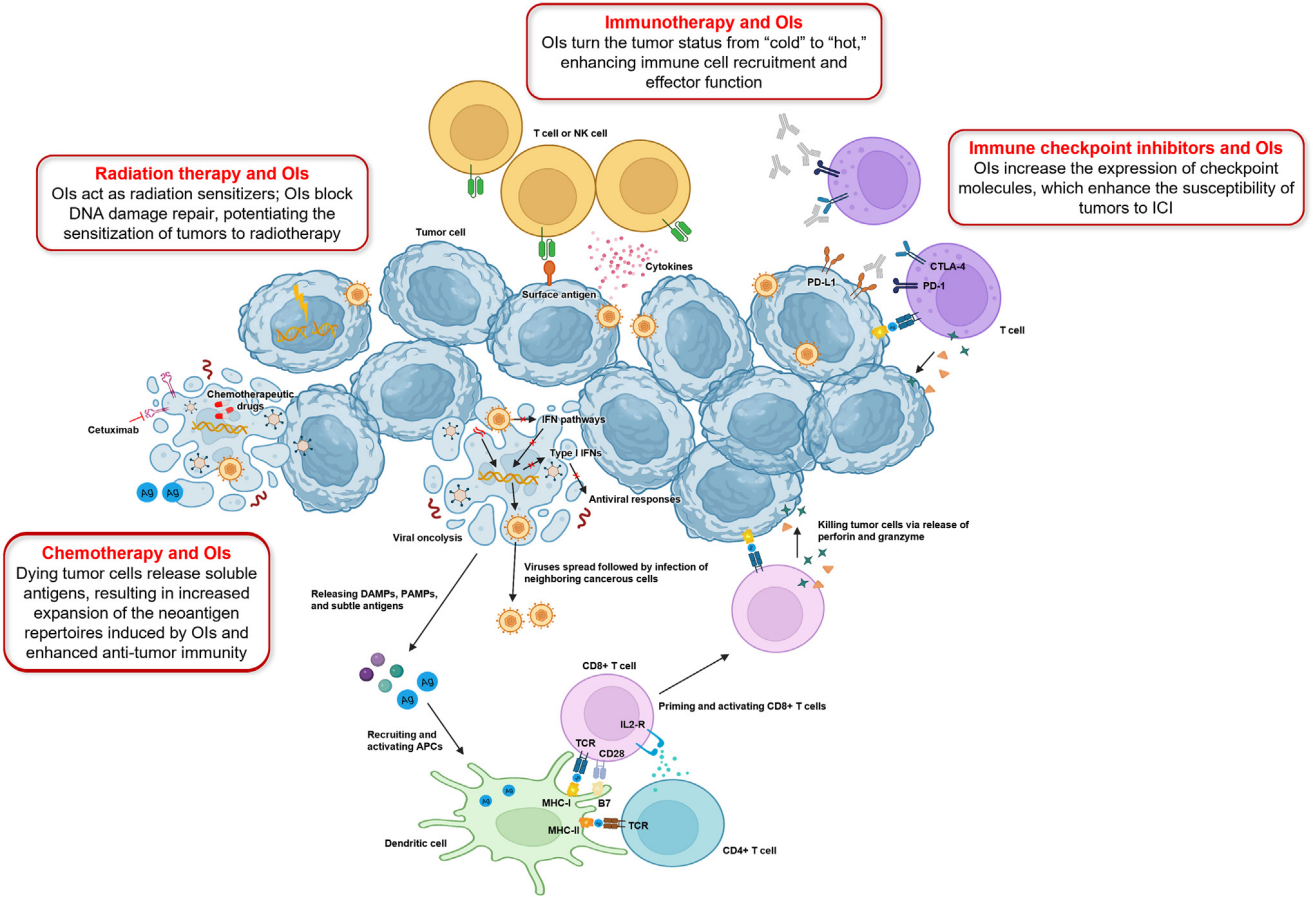
Oncolytic virotherapy as a combined strategy to potentiate anti-tumor efficacy Adapted from *Molecular Cancer*, volume 19, issue 158, Zhang B and Cheng P. Improving anti-tumor efficacy via combinatorial regimens of oncolytic virotherapy, Copyright 2020, with permission from Springer Nature under CC BY 4.0 license. https://molecular-cancer.biomedcentral.com/articles/10.1186/s12943-020-01275-6. APC, antigen-presenting cell; B7, CTLA-4 counter-receptor; CD, cluster of differentiation; CTLA-4, cytotoxic T-lymphocyte antigen 4; DAMP, damage-associated molecular pattern; ICI, immune checkpoint inhibitor; IFN, interferon; IL2-R, interleukin-2 receptor; MHC, major histocompatibility complex; NK, natural killer; OI, oncolytic immunotherapy; PAMP, pathogen-associated molecular pattern; PD-L1, programmed cell death-ligand 1; TCR, T cell receptor.

Non-overlapping toxicities and the potential for mechanistic synergy create opportunities for OI combinations with other cancer therapeutics such as chemotherapy and radiotherapy (Figure 5).^8^^,^^16^ For example, combining chemotherapy and OIs has been shown to enhance the induction of apoptosis by cisplatin and paclitaxel *in vitro*.^87^^,^^88^ Damage of tumor cells exposed to chemotherapy and OIs may lead to more efficient release of soluble antigens, potentially leading to enhanced anti-tumor immunity.^1^^,^^8^ OIs also block DNA damage repair, thereby potentiating the sensitization of tumors to radiotherapy.^1^^,^^8^ Due to their tumor tropism, OIs can also facilitate the accumulation of radionuclides in tumor cells, increasing the precision and safety of radiation treatments.^8^ Additionally, adoptive T cell therapy approaches may also benefit from the addition of OIs, which promote the expression of major histocompatibility complex molecules, enabling tumor-infiltrating lymphocyte and engineered T cell receptor therapies to infiltrate and kill tumors.^8^^,^^89^ The significance and how best to implement these novel combinations of OIs into clinical practice will require reassessment when additional safety and efficacy data become available from ongoing clinical trials, such as those evaluating TBI-1401 and chemotherapy (NCT03252808) or G207 and radiation (NCT04482933).

## Utilizing OI as monotherapy

An opportunity for OIs to be used as monotherapy may exist in patients for whom ICIs are often contraindicated or not well tolerated. For example, patients undergoing solid organ transplantation require chronic immunosuppression, which increases their risk of developing skin cancer, including cutaneous squamous cell carcinoma and melanoma.^90^ The use of ICIs has dramatically improved outcomes in advanced skin cancers in immunocompetent individuals,^91^ but their use in solid organ transplant recipients warrants great caution given the significant risk of allograft rejection.^92^ In addition, ICIs may have the potential to trigger or exacerbate autoimmune disorders by removing the physiological inhibition that normally prevents autoreactivity.^93^^,^^94^ Several case reports have documented the safety and efficacy of T-VEC in solid organ transplant recipients with melanoma.^95^^,^^96^^,^^97^ RP1 monotherapy is also being evaluated for safety and efficacy in solid organ and hematopoietic cell transplant recipients with advanced or metastatic cutaneous malignancies (ARTACUS; NCT04349436).^98^ Initial data from the first 13 kidney transplant recipients (12 with cutaneous squamous cell carcinoma and one with Merkel cell carcinoma) showed an ORR of 27.3% (all complete responses); RP1 appeared to be well tolerated and no evidence of allograft rejection was seen.^98^

## Challenges and considerations

Therapy with OIs requires several unique considerations. Since OIs are derived from live and/or attenuated viruses, virus design must be carefully considered before determining whether an OI may be appropriate for human use. Immunocompromised individuals, such as patients with human immunodeficiency virus, those with past hepatitis B/C infection, and those on immunosuppressive therapies, may be at risk for disseminated viral spread, depending on the virus used and degree of immunosuppression.^99^ Furthermore, the immune response is a double-edged sword. In part, the efficacy of OIs is dependent on triggering a robust immune response, but the same immune response also has the ability to limit OI spread. This is especially relevant with regard to the debate concerning whether OIs should be administered intravenously or intratumorally.^50^ While intravenous administration offers several benefits, such as the simplicity of the delivery method and the possibility for the OI to reach distant sites of metastatic disease, there is concern with intravenously administered OIs that pre-existing neutralizing antibodies from natural exposure to viral strains or the development of neutralizing antibodies from repeated OI infusions could limit the efficacy of OIs.^100^^,^^101^ For example, intravenous administration of HSV1716, an HSV-1-based OI, induced an antiviral immune response in all patients for whom data were available. While the patients developed PCR-positive viremia after the first OI injection, they were PCR negative after subsequent injections, and the antiviral immune response of the patients was likely a contributing factor.^102^ Beyond susceptibility to antiviral immunity, there is also concern that intravenously administered OIs may be limited by rapid dilution of the OI in systemic circulation or suffer from sequestration in the fenestrated capillaries of the lung, spleen, and liver.^1^^,^^100^

The use of carrier cells represents one potential approach to shield the OI from neutralizing antibodies during intravenous administration. There are several cell types used in this capacity, such as mesenchymal stem cells and immune cells, both of which have been reviewed previously.^103^^,^^104^ Briefly, autologous patient-derived carrier cells are loaded with the OI *ex vivo*, prior to intravenous injection. The OI-infected carrier cells then migrate to the tumor microenvironment, effectively shielding the OI from neutralizing antibodies in the bloodstream. To be effective, carrier cells must exhibit tumor tropism and maintain viability long enough in the tumor microenvironment to support replication and release of viral progeny to infect the tumor.^103^^,^^104^ This strategy may be particularly useful in HSV-based OIs where the seroprevalence of HSV is high.^104^ However, this approach remains largely at the stage of preclinical development, with few clinical trials evaluating the use of carrier cells (NCT02068794, NCT01844661, NCT03896568, NCT05047276, and NCT0307213).^103^^,^^104^

Strategies are also being explored to minimize the potential downsides of intravenously administered OIs using alternative viral species. For example, the vaccinia virus is being pursued as an OI (JX-594; NCT02630368) for intravenous delivery. This strategy is supported by the unique biology of the virus that includes production of extracellular enveloped viruses that can evade neutralizing antibodies.^105^ However, PCR or immunohistochemistry detection of the OI within biopsied tumors only occurred at the highest dose tested of 10^9^ plaque-forming units per dose. The possibility that such a high dose saturated the inhibitory potential of any antiviral immune response cannot be ruled out.^105^ Alternatively, to circumvent neutralizing antibodies, the development of OIs encapsulated in lipid nanoparticles is being explored at the preclinical stage but awaits further clinical validation.^106^ While intratumoral administration of OIs overcomes the challenges of antibody neutralization and offers tumor-specific delivery of high viral loads,^1^^,^^50^^,^^107^ it generates a different hurdle for a requirement of at least one injectable tumor (e.g., ≥1 cm in longest diameter). This has been less of an issue with recent studies taking advantage of image guidance to access deep lesions and the incorporation of potent immune-stimulating transgenes into novel OIs to maximize the chances of systemic immune stimulation, thus obviating systemic delivery.^14^ However, logistical constraints around repeated image-guided delivery of OIs to deep tissue or visceral lesions and a lack of standardized delivery techniques present hurdles that will need to be addressed in order to optimize outcomes for patients receiving intratumoral OIs.^108^^,^^109^ Furthermore, as the OI field extends beyond superficial tumors, new safety concerns may arise. For example, many clinical protocols for OI delivery require weekly or biweekly injections, and thus the risk of injury from repeated needle punctures in organs such as the liver will need to be assessed. Also, the full impact of repeated injections in highly vascularized organs, compared with the dermis, on increasing systemic toxicities warrants further attention.

While it should be noted that the majority of OIs in clinical development use intratumoral delivery, there is a lack of studies directly comparing the efficacy of intravenous versus intratumoral administration, and thus the topic warrants further investigation. In this regard, T3011 is currently being evaluated for intravenous administration in two separate phase 1/2a clinical trials in advanced solid tumors (NCT05598268 and NCT04780217). The results from these clinical trials may be of particular interest as there are also ongoing clinical trials evaluating T3011 for intratumoral injection in advanced solid tumors (NCT05602792 and NCT04370587). While these clinical trials are not intended to be a head-to-head evaluation of intravenous versus intratumoral injection, the use of the same OI agent in similar tumor types has the potential to yield the most relevant clinical efficacy data to date comparing intravenous versus intratumoral injection.

## Conclusions

OI, a novel and promising option in cancer treatment, offers considerable flexibility and can be enhanced by genetic engineering to improve efficacy and safety. While effective as standalone agents in some circumstances, OIs may work most effectively in combination with other systemic therapeutics. Progress in the field will depend on fine-tuning the balance of development of innovative strategies to boost anti-tumor-directed immunity while also optimizing combinations with other drugs.

## Supplemental information

Document S1. Table S1

Document S2. Article plus supplemental information
