# Supplementary material for: Breaking the barriers in cancer care: The next generation of herpes simplex virus-based oncolytic immunotherapies for cancer treatment
Source: Mol Ther Oncolytics. 2023 Sep 19;31:100729. doi: 10.1016/j.omto.2023.100729 (PMC10570124; doi:10.1016/j.omto.2023.100729)
Supplement: Document S1. Table S1 [file mmc1.pdf]

OMTO, Volume *31*

Supplemental information

Breaking the barriers in cancer care: The next generation of herpes simplex virus-based oncolytic immunotherapies for cancer treatment

Nikhil I. Khushalani, Kevin J. Harrington, Alan Melcher, Praveen K. Bommareddy, and Dmitriy Zamarin

Supplemental Material

Table S1. Differential properties of select HSV–based OIs.

	**T-VEC[1]**	**TBI-1401[2]**	**G207[3]**	**G47Δ[4]**	**T3011[5]**	**ONCR-177[6]**	**RP3[a]**	**OH2[7]**
**HSV serotype**	HSV-1	HSV-1	HSV-1	HSV-1	HSV-1	HSV-1	HSV-1	HSV-2
**Genome**	dsDNA							
**Genome size**	Large (152 kb)							
**Cell entry mechanism**	Endocytosis; penetration							
**Cell entry receptor**	HVEM and nectin-1							
**Transgene capacity**	High							
**Viral immunogenicity**	Low							
**Pathogenicity of native virus**	Cold sore, fever blister, possibility of latent infection in CNS							
**Strategies for attenuating pathogenicity**	Deletion of ICP34.5 and ICP47 genes; modified US11 promoter	Naturally occurring deletion of UL56 and Latency-associated transcript	Deletion of ICP34.5 and ICP6	Deletion of ICP34.5, ICP6, and ICP47 genes; modified US11 promoter	Not reported	Mutated US12 and UL37 with miRNA targeting of ICP4, ICP27, UL8, and ICP34.5	Deletion of ICP34.5 and ICP47 genes; modified US11 promoter	Deletion of ICP34.5 and ICP47 genes; modified US11 promoter
**Insertions**	GM-CSF	-	-	-	IL-12<br>αPD-1	αCTLA-4<br>αPD-1<br>ECD of FLT3LG<br>CCL4<br>IL-12	GALV-GP-R–<br>αCTLA-4<br>CD40L<br>4-1BBL	GM-CSF

[a]RP1 and RP2 are OIs derived from the same backbone as RP3; RP1 contains only GALV-GP-R– and GM-CSF; RP2 contains the RP1 modifications plus the addition of αCTLA-4. Properties shared by all the OIs presented are listed only once in the middle of the table.

4-1BBL, 4-1BB ligand; αCTLA-4, anti–cytotoxic T-lymphocyte antigen 4; αPD-1, anti–programmed cell death protein 1; CCL4, C-C motif chemokine ligand 4; CNS, central nervous system; CD40L, cluster of differentiation 40 ligand; dsDNA, double-stranded DNA; ECD of FLT3LG, extracellular domain of Fms related receptor tyrosine kinase 3 ligand; GALV-GP-R–, gibbon ape leukemia virus glycoprotein with the R sequence deleted; GM-CSF, granulocyte-macrophage colony-stimulating factor; HSV-1, herpes simplex virus type 1; HVEM, herpes virus entry mediator; ICP, infected cell protein; IL-12, interleukin-12; kb, kilobase; miRNA, microRNA; OI, oncolytic immunotherapy; T-VEC, talimogene laherparepvec; UL, unique long region; US, unique short sequence.

References

1. Hu, J.C., Coffin, R.S., Davis, C.J., Graham, N.J., Groves, N., Guest, P.J., Harrington, K.J., James, N.D., Love, C.A., McNeish, I., et al. (2006). A phase I study of OncoVEXGM-CSF, a second-generation oncolytic herpes simplex virus expressing granulocyte macrophage colony-stimulating factor. Clin Cancer Res. *12*, 6737-6747. 10.1158/1078-0432.Ccr-06-0759.
2. Eissa, I.R., Naoe, Y., Bustos-Villalobos, I., Ichinose, T., Tanaka, M., Zhiwen, W., Mukoyama, N., Morimoto, T., Miyajima, N., Hitoki, H., et al. (2017). Genomic signature of the natural oncolytic herpes simplex virus HF10 and its therapeutic role in preclinical and clinical trials. Front Oncol. *7*, 149. 10.3389/fonc.2017.00149.
3. Cripe, T.P., Chen, C.Y., Denton, N.L., Haworth, K.B., Hutzen, B., Leddon, J.L., Streby, K.A., Wang, P.Y., Markert, J.M., Waters, A.M., et al. (2015). Pediatric cancer gone viral. Part I: strategies for utilizing oncolytic herpes simplex virus-1 in children. Mol Ther Oncolytics. *2*, 15015-. 10.1038/mto.2015.15.
4. Todo, T., Martuza, R.L., Rabkin, S.D., and Johnson, P.A. (2001). Oncolytic herpes simplex virus vector with enhanced MHC class I presentation and tumor cell killing. Proc Natl Acad Sci U S A. *98*, 6396-6401. 10.1073/pnas.101136398.
5. Haydon, A., Kichenadasse, G., Kirkwood, J., Kaufman, H., Buchbinder, E., Ganju, V., Barve, M., Jiang, H., Xu, H., Zhou, X., et al. (2021). A phase 1, open-label, dose escalation study of the safety and tolerability of T3011 in advanced cutaneous or subcutaneous malignancies. J Clin Oncol. *39*, 2526-2526. 10.1200/JCO.2021.39.15_suppl.2526.
6. Haines, B.B., Denslow, A., Grzesik, P., Lee, J.S., Farkaly, T., Hewett, J., Wambua, D., Kong, L., Behera, P., Jacques, J., et al. (2021). ONCR-177, an oncolytic HSV-1 designed to potently

activate systemic antitumor immunity. Cancer Immunol Res. *9*, 291-308. 10.1158/2326-6066.Cir-20-0609.

7. Zhang, B., Huang, J., Tang, J., Hu, S., Luo, S., Luo, Z., Zhou, F., Tan, S., Ying, J., Chang, Q., et al. (2021). Intratumoral OH2, an oncolytic herpes simplex virus 2, in patients with advanced solid tumors: a multicenter, phase I/II clinical trial. J Immunother Cancer. *9*, e002224. 10.1136/jitc-2020-002224.
